# Conventional transcutaneous electrical nerve stimulation shows no short-term safety signal in painful cervical dystonia: a pilot prospective study

**DOI:** 10.3389/fneur.2026.1909821

**Published:** 2026-10-07

**Authors:** Jason Becker, Jonathan Epstein, Solène Frismand, Salomé Puisieux

**Affiliations:** 1 Department of Neurology, University Hospital of Nancy, Nancy, France; 2 Department of Clinical Epidemiology, INSERM, University of Lorraine and University Hospital of Nancy, Nancy, France; 3 NGERE, INSERM, University of Lorraine, Nancy, France

**Keywords:** botulinum toxin, cervical dystonia, neuromodulation, pain, pilot study, transcutaneous electrical nerve stimulation, TWSTRS

## Abstract

**Background and purpose:**

Cervical dystonia (CD) is frequently associated with chronic cervical pain refractory to botulinum neurotoxin (BoNT) therapy. Transcutaneous electrical nerve stimulation (TENS), a non-invasive neuromodulation technique with established analgesic properties, has not been formally evaluated in CD. This pilot study assessed the safety and tolerability of conventional TENS in painful CD, with exploratory evaluation of pain outcomes.

**Methods:**

This prospective, observational, single-arm study (NCT04949594) included patients with painful CD treated or not with BoNT. The primary outcome was the mean change in Toronto Western Spasmodic Torticollis Rating Scale (TWSTRS) total score between peak BoNT efficacy time points before (T1) and after TENS introduction (T2), assessed against an exploratory −8-point safety threshold. Secondary outcomes included TWSTRS subscores and Pain Numeric Rating Scale (PNRS).

**Results:**

Fifteen patients completed the study. The mean T1–T2 TWSTRS total score change was +3.42 (95% CI − 0.86 to 7.69), remaining above the exploratory −8-point safety threshold, with no group-level signal of clinically meaningful worsening. At the individual level, only three patients (20%) showed a worsening, two of whom (13.3%) exceeded the −8-point threshold (−11.5 and −9.5 points). TWSTRS subscores and PNRS showed no statistically significant changes after correction for multiple comparisons. Adverse events were mild and transient, and 80% of patients reported satisfaction and continued TENS use at follow-up.

**Conclusion:**

Conventional TENS showed no short-term safety signal and was well tolerated in painful CD, supporting its feasibility as an adjunctive neuromodulation approach in this small, uncontrolled pilot cohort, pending confirmation in randomized sham-controlled trials.

## Introduction

Cervical dystonia (CD) is the most frequent form of focal dystonia in adults and is characterized by sustained or intermittent involuntary contractions of cervical muscles, resulting in abnormal head postures and movements (1, 2). Beyond motor impairment, pain represents one of the most prominent and disabling non-motor features of the disorder. Chronic neck pain affects up to 80–90% of patients and is a major determinant of reduced quality of life, psychological burden, and healthcare utilization (2).

Botulinum toxin (BoNT) injections are the first-line treatment for cervical dystonia and effectively reduce abnormal muscle activity (3, 4). Although BoNT often improves pain, a substantial proportion of patients continue to experience clinically significant pain despite optimized treatment, making persistent pain one of the major unmet therapeutic needs in CD (4).

Pain in CD is multifactorial and cannot be explained solely by peripheral muscle overactivity. Increasing evidence supports dystonia as a disorder of abnormal sensorimotor integration, impaired inhibitory control, and maladaptive plasticity within cortical and subcortical networks (2, 4). These mechanisms are closely linked to altered sensory processing and may contribute both to dystonic motor output and pain perception, providing a rationale for sensory-based neuromodulatory interventions.

Transcutaneous electrical nerve stimulation (TENS) is a non-invasive neuromodulation technique widely used for acute and chronic pain of various etiologies. Large meta-analyses have demonstrated that TENS produces clinically meaningful analgesia largely independent of the underlying diagnosis, with a favorable safety profile (5, 6). While TENS has not been formally evaluated in CD, its established effects on cervical pain, central pain modulation, and sensorimotor pathways suggest potential relevance for patients with painful CD (7).

The primary objective of this study was to assess the safety and tolerability of conventional TENS in patients with CD, with secondary exploratory assessment of pain-related outcomes and patient-reported benefit.

## Materials and methods

### Study design

TENDYS (TENs use in CD) is an observational, prospective, single-arm, monocentric study conducted between September 2019 and July 2020 with CD treated or not with BoNT A at the Nancy Regional University Hospital (ClinicalTrials.gov: NCT04949594). This study was designed as a pilot prospective observational safety and feasibility study, aiming primarily to ensure that conventional TENS would not induce clinically meaningful worsening of dystonia severity.

All patients with painful CD receiving standard follow-up or BoNT injections every 3 months were offered TENS during a baseline neurological consultation (T0). Patients who declined TENS were excluded. Those who accepted provided informed consent and were reassessed approximately one month after botulinum toxin injection, corresponding to peak treatment efficacy, to confirm persistent pain and receive a TENS demonstration (T1). Patients were then reassessed approximately one month after their next botulinum toxin injection (T2), ensuring that T1 and T2 evaluations were performed at comparable time points within the botulinum toxin treatment cycle.

The final study protocol and the informed consent process were approved by the Investigation and Clinical Research Committee of the University Medical Center of Nancy, France.

### Population

Patients with CD were eligible for inclusion if they had idiopathic CD, were older than 18 years, treated or untreated with BoNT, and presented with pain attributable to CD that was considered clinically significant (PNRS ≥ 3 and reported by the patient as meaningfully disabling). Attribution of pain to CD was based on clinical examination, correlating pain location and pattern with the dystonic posture and, when available, imaging; patients whose pain was judged exclusively degenerative or musculoskeletal were not included. Because cervical pain in this population is frequently multifactorial, a mixed contribution could not always be excluded. Exclusion criteria were pregnancy, inability to follow study procedures, legal guardianship, and pacemaker implantation, in line with TENS contraindications (5).

For patients receiving BoNT, injected muscles and dosing were determined at the physician’s discretion. Concomitant treatments for focal dystonia were allowed if kept at a stable dose. Patient demographics and CD characteristics were obtained from the DxCare medical record system or clinical examination.

### TENS protocol

Electrical stimulation (Cefar TENS (Chattanooga®) or TENS ECO2, Schwa-Medico®) was applied to the posterior cervical muscles over or near the painful area using self-adhesive electrodes. Stimulation consisted of an asymmetric biphasic rectangular waveform at 80–100 Hz with a pulse width of 180 μs (conventional high-frequency/low-intensity, gate control mode). Intensity was set approximately 1.5 mA below the pain and motor thresholds, producing a tingling sensation without muscle contraction. Patients used TENS daily according to tolerance, outside sleeping and driving periods. Electrode placement and stimulation waveform, frequency and pulse width were standardized across both devices and were not modified during follow-up; only stimulation intensity could be adjusted by the patient, within the pre-specified sub-motor-threshold range, to maintain a comfortable tingling sensation. Daily use duration and adherence were captured by patient self-report at the T2 visit (Supplementary Table 1 and 2).

### Outcome measures

The primary outcome was the mean change in the TWSTRS-total score between T1 and T2. Secondary outcomes included mean changes in TWSTRS Severity, Disability and Pain subscores (8–10), and PNRS scores from T1 to T2. At the final visit (T2), patients completed a standardized satisfaction survey. The TWSTRS is a disease-specific scale comprising three subscales: severity (0–35), disability (0–30), and pain (0–20), yielding a total score from 0 to 85 (8–10). Pain intensity was assessed using a 0–10 PNRS (11).

### Statistical analysis

The −8-point threshold was selected as an exploratory safety threshold based on published anchor-based estimates of the minimal clinically important change (MCIC) in two independent cohorts. Dashtipour et al. (12) identified an MCIC of −8 points for “minimal improvement” based on Patient Global Impression of Change (PGIC) ratings in 479 patients from the CD PROBE registry. Espay et al. (13) reported an MCIC of −11.9 points in 304 patients with a mean baseline TWSTRS-total score of 43.4, with thresholds ranging from −3.2 to −18.0 depending on baseline severity. As no direction-specific threshold for clinically meaningful worsening has been established for the TWSTRS to our knowledge, we used the more conservative of the two published estimates (−8 points, the smaller magnitude) as an exploratory safety threshold, assuming that a change of similar magnitude in the opposite direction would be clinically meaningful. This assumption has not been formally validated.

Change was defined as the T1 minus T2 score, with negative values indicating worsening (higher score at T2) and positive values indicating improvement or stability. No group-level signal of clinically meaningful worsening was detected, as the lower bound of the 95% confidence interval for the mean T1–T2 change remained above the exploratory −8-point safety threshold. This threshold was used for qualitative interpretation of the group-level results and was not considered a formally validated non-inferiority margin.

Wilcoxon signed-rank tests were used to assess within-group changes. Secondary outcomes were analyzed descriptively. Statistical significance was set at *p* < 0.05.

## Results

### Characteristics of CD population

A total of 20 patients with painful CD were included in the TENS cohort. Their mean age was 55.8 ± 12.9 years, with a mean age at symptom onset of 44.3 ± 12.1 years (Table 1). Most participants were women (65%). Dystonia was focal in 60% of cases, segmental in 20%, multifocal in 15%, and generalized in 5%. All patients reported clinically significant cervical pain, with a mean PNRS score of 5.9 ± 1.7 (range 3–10). At baseline, pain intensity was rated as mild in 5% of patients, moderate in 60%, and severe in 35%.

**Table 1:** Baseline characteristics of patients with painful cervical dystonia included in the TENDYS study (*n* = 20).

Characteristic	*n* = 20
Demographics	
Age, years — Mean ± SD	55.8 ± 12.9
Age at symptom onset, years — Mean ± SD	44.3 ± 12.1
Female sex, *n* (%)	13 (65)
BMI, kg/m^2^ — Mean ± SD	23.6 ± 4.3
Cervical dystonia characteristics	
CD distribution, *n* (%)	
Focal	12 (60)
Segmental	4 (20)
Multifocal	3 (15)
Generalized	1 (5)
Cervical pain, *n* (%)	20 (100)
PNRS score — Mean ± SD [range]	5.9 ± 1.7 [3–10]
Botulinum neurotoxin therapy	
BoNT-treated patients, *n* (%)	17 (85)
Onabotulinumtoxin A (BOTOX®)	13 (65)
Incobotulinumtoxin A (XEOMIN®)	3 (15)
Abobotulinumtoxin A (DYSPORT®)	1 (5)
Total dose injected, U — Mean ± SD	171.3 ± 59.5
Injection interval, months — Mean ± SD	2.9 ± 0.4
Treatment duration, years — Mean ± SD	5.2 ± 7.1
No BoNT injection, *n* (%)	3 (15)
Concomitant antidystonic medication	
Any antidystonic medication, *n* (%)	6 (30)
Benzodiazepines	5 (83.3)
Dopaminergics	1 (16.7)
Antidopaminergics	0
Anticholinergics	1 (16.7)
Baclofen	0
Combination	2 (33.3)
Physiotherapy	
Physiotherapy with a physiotherapist, *n* (%)	8 (40)
Sessions per month — Mean ± SD	6 ± 2.8
Self-directed physiotherapy exercises, *n* (%)	6 (30)

BMI, body mass index; BoNT, botulinum neurotoxin; CD, cervical dystonia; PNRS, Pain Numeric Rating Scale. Percentages for antidystonic medication sub-categories are calculated among the 6 patients receiving such medication.

17 patients (85%) were receiving BoNT injections, while three (15%) were not. Onabotulinumtoxin A (BOTOX®) was the most frequently used formulation (13/20, 6%), followed by incobotulinumtoxin A (XEOMIN®) (3/20, 15%) and abobotulinumtoxin A (DYSPORT®) (1/20, 5%). The mean injected dose was 171.3 ± 59.5 U, with a mean interval between injections of 2.9 ± 0.4 months and a mean treatment duration of 5.2 ± 7.1 years. Concomitant antidystonic medication was used by 30% of patients, and 40% were receiving physiotherapy at a mean frequency of 6 ± 2.8 sessions per month. Half also performed self-directed physiotherapy exercises. Injected-muscle selection was unchanged between the T1 and T2 BoNT cycles for all treated patients. Total BoNT dose was unchanged between the 2 cycles in 6 of the 12 BoNT-treated completers and was modified (increased or decreased at the treating physician’s discretion) in the remaining 6.

Among the 20 included patients, three had previously used TENS for cervical pain before study initiation and were retained for descriptive analyses only. 17 patients were therefore prescribed TENS. Two subsequently withdrew: one due to spontaneous relief of cervical pain, and one due to study-related constraints. Data from the remaining 15 patients were analyzed for primary and secondary outcomes (Figure 1).

**Figure 1:**
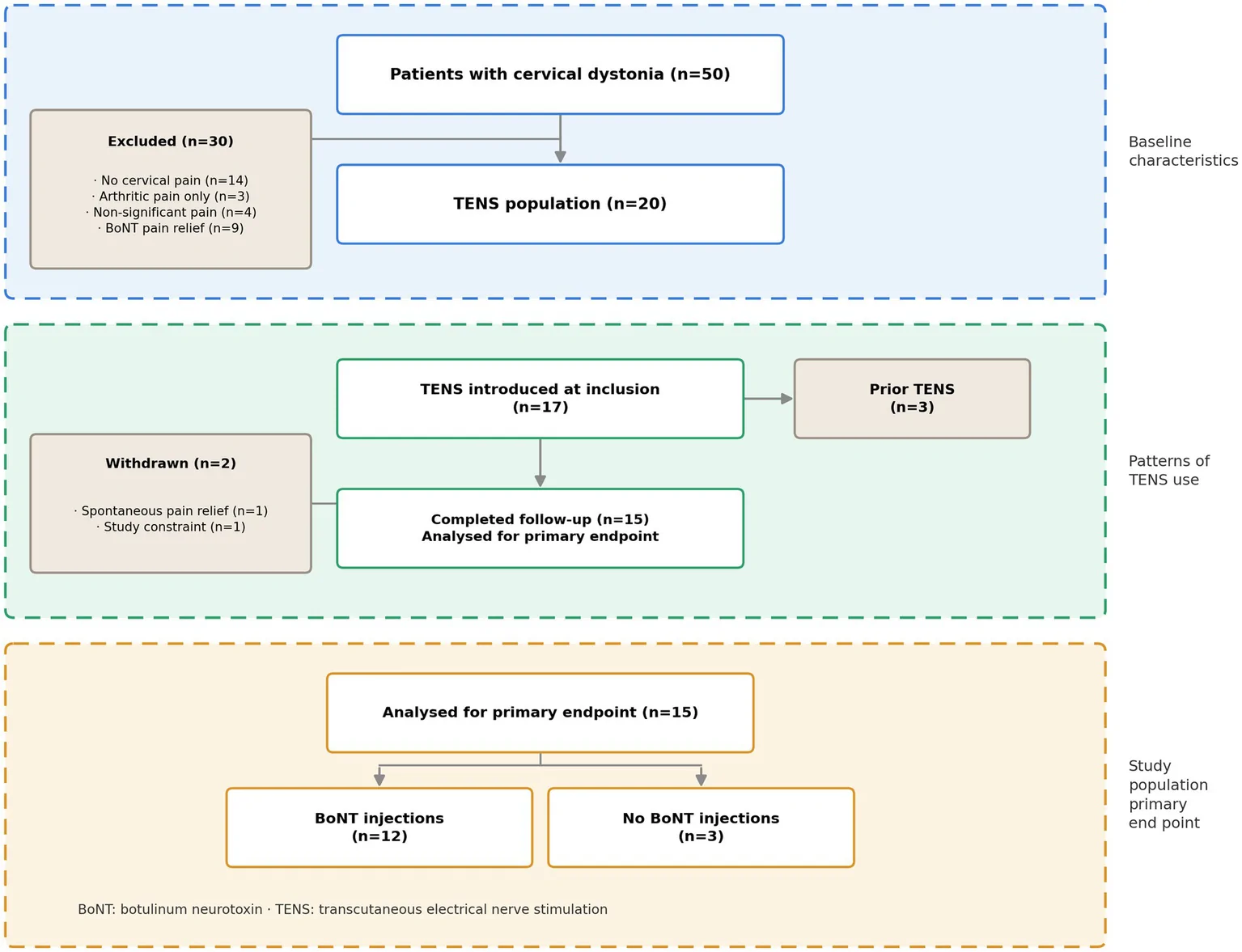
Flow Chart.

### Primary outcome

The TWSTRS total score did not show clinically meaningful worsening following introduction of TENS. The mean T1–T2 difference was +3.42 (SD 7.72; 95% CI: −0.86 to 7.69; median +3.00, IQR 6.88). The lower bound of the confidence interval did not cross the predefined exploratory −8-point safety threshold, supporting the short-term safety of conventional TENS regarding dystonia severity at the group level (Figure 2). At the individual level, only three of the 15 patients (20%) had a negative T1–T2 change (i.e., an individual worsening); two of these three (13.3% of the cohort) fell below the predefined exploratory −8-point threshold (−11.5 and −9.5 points, respectively), while the third had a smaller worsening of −2.5 points. The remaining 12 patients (80%) showed stability or improvement (T1–T2 ≥ 0). These individual trajectories are displayed in Figure 2.

**Figure 2:**
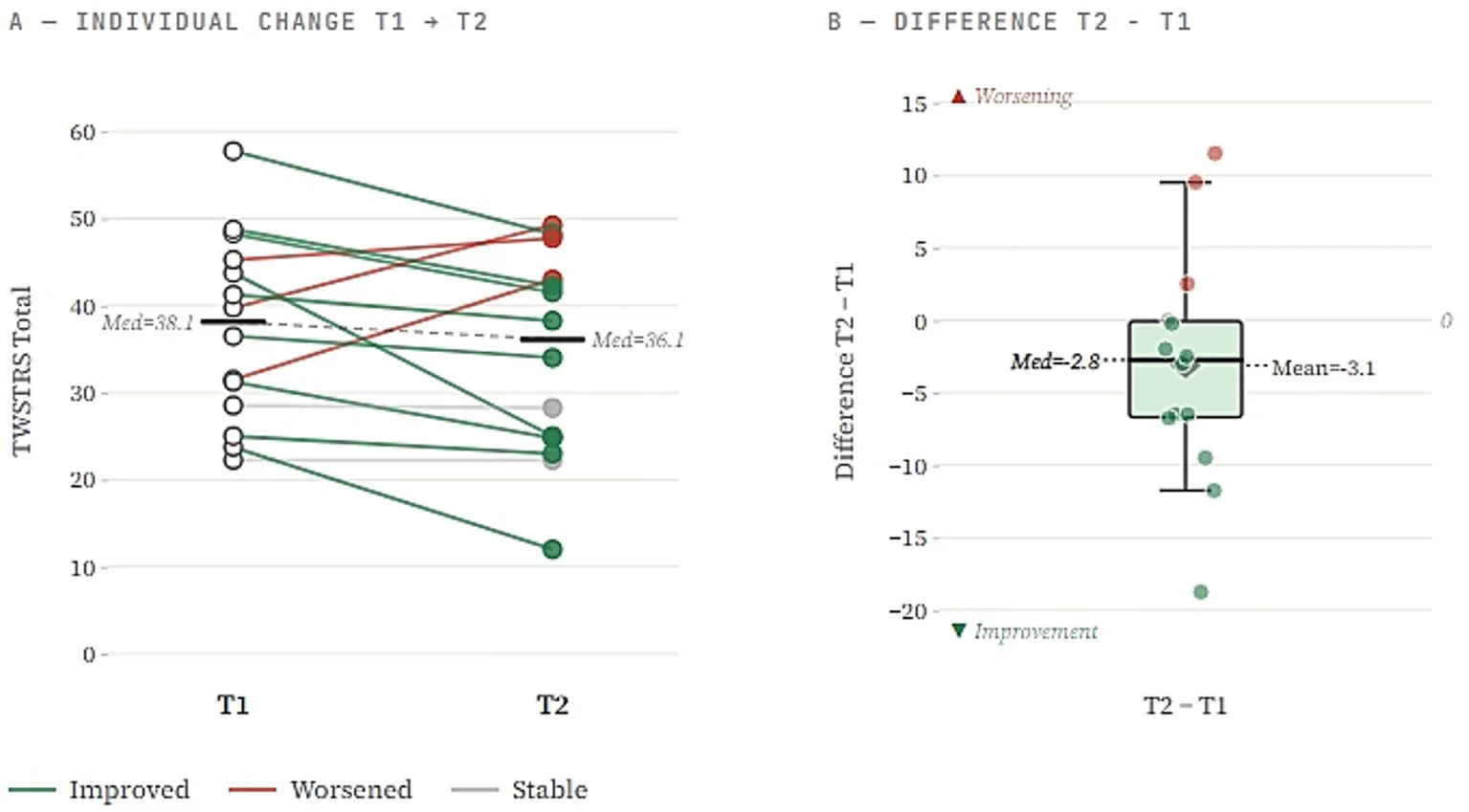
TWSTRS Total score at T1 and T2 in subjects who completed study (*n* = 15). **(A)** Each line represents the change in TWSTRS Total score for one subject between T1 (peak BoNT efficacy without TENS) and T2 (peak BoNT efficacy with TENS). Green lines indicate improvement (lower score at T2); red lines indicate worsening. Horizontal tick marks show the group median at each time point. **(B)** Distribution of the difference T1 − T2 (positive values = improvement, negative values = worsening; same sign convention as used throughout the manuscript). The central line represents the median; the box spans the interquartile range (Q1–Q3); whiskers extend to the observed minimum/maximum excluding outliers. Individual data points are overlaid with horizontal jitter. The diamond represents the mean. The dashed line marks zero (no change). T1: peak BoNT efficacy without TENS; T2: peak BoNT efficacy with TENS. TWSTRS: Toronto Western Spasmodic Torticollis Rating Scale.

### Secondary outcomes

TWSTRS Severity showed a nominal improvement between T1 and T2 (+2.07 ± 3.58; Wilcoxon signed-rank test, raw *p* = 0.046), which did not remain statistically significant after Holm-Bonferroni correction for the four comparisons in Table 2 (adjusted *p* = 0.182). Disability (+0.20 ± 4.48) and Pain (+1.15 ± 3.67) subscores, and PNRS (+0.1 ± 2.7), none remained statistically significant after Holm–Bonferroni correction (Table 2).

**Table 2:** Changes in TWSTRS subscores and PNRS between T1 and T2 in patients who completed the study (*n* = 15).

Score (T1–T2 difference)	Mean ± SD [range]	Median [IQR]	*p* (raw)	*p* (adjusted)*
TWSTRS-Severity	2.07 ± 3.58 [−3.0–10.0]	2.0 [4.0]	0.046	0.182
TWSTRS-Disability	0.20 ± 4.48 [−8.0–9.0]	0.0 [5.5]	0.752	1.000
TWSTRS-Pain	1.15 ± 3.67 [−4.5–6.75]	1.5 [5.1]	0.248	0.744
PNRS	0.1 ± 2.7 [−5.0–5.0]	0 [3.5]	0.850	1.000

PNRS, Pain Numeric Rating Scale; TWSTRS, Toronto Western Spasmodic Torticollis Rating Scale; IQR, interquartile range. Values are T1–T2 differences; positive values indicate improvement (score decrease) and negative values indicate worsening (score increase). *p (adjusted) uses the Holm-Bonferroni correction across the four comparisons in this table. TWSTRS Severity showed a nominal improvement (raw *p* = 0.046) that did not remain statistically significant after correction for multiple comparisons (adjusted *p* = 0.182); none of the changes in this table reached statistical significance after correction.

### Sensitivity analysis: dose-stable subgroup

To assess whether BoNT dose changes might have contributed to the primary outcome, the analysis was repeated in the subgroup of patients with an unchanged BoNT dose between T1 and T2, plus the non-BoNT patients (*n* = 9: 6 dose-stable BoNT-treated + 3 non-BoNT). The mean T1–T2 TWSTRS-total change was +4.44 (SD 7.98; 95% CI − 1.69 to 10.58; median +3.00, IQR 7.00; Wilcoxon *p* = 0.109), and the mean PNRS change was +1.03 (95% CI − 0.26 to 2.32; median +2.00; *p* = 0.125). The lower bound of the TWSTRS-total confidence interval remained well above the predefined −8-point exploratory safety threshold, and none of the changes reached statistical significance, consistent with the primary analysis of the full *n* = 15 cohort. These findings suggest that changes in BoNT dose are unlikely to explain the absence of a safety signal. This subgroup analysis should be interpreted as a sensitivity analysis showing results in a similar direction to those observed in the full cohort, rather than as evidence excluding BoNT-related confounding: given the small sample size (*n* = 9) and the *post hoc* nature of the analysis, residual confounding related to BoNT cannot be ruled out.

### TENS use pattern and tolerability

Unless otherwise specified, all percentages reported in this section are calculated on the 15 patients who completed follow-up and were analyzed for primary and secondary outcomes (Figure 1). Among patients who completed the study, mean daily TENS use was 72.3 ± 42.3 min (range 30–150 min) (Supplementary Table 1). Pain relief onset occurred within a mean of 5.3 ± 9.1 min after electrode application. A residual analgesic effect lasting at least one hour after TENS removal was reported by 80% of patients (12/15), and mean self-reported pain relief was 29.5 ± 20.6% (range 0–60%), reflecting marked inter-individual variability in response. At the end of follow-up, 80% of patients (12/15) reported overall satisfaction and expressed willingness to continue TENS use (Supplementary Table 1).

Adverse events were reported in 40% of patients (6/15). All were mild and transient: three patients reported electrode detachment with local discomfort, resolved with conductive adhesive gel or electrode repositioning; two reported occasional electrical shocks at electrode detachment; and one reported shoulder and arm fatigue occurring 2–3 days after each session, which ultimately led to discontinuation despite reported analgesic efficacy. No allergic reaction was recorded (Supplementary Table 2).

Three patients discontinued TENS during follow-up: one due to cervical pain predominantly attributable to osteoarthritis with limited TENS benefit on this non-dystonic component; one due to shoulder and arm fatigue as described above; and one who reported spontaneous pain improvement with physiotherapy alone and did not perceive additional benefit from TENS (Supplementary Table 2). These three patients discontinued TENS itself but remained in the study and are part of the analyzed *n* = 15 cohort, with observed T2 TWSTRS data, distinct from the two patients who withdrew from the study altogether prior to the primary analysis. Of note, some patients reported subjective benefit — including muscle relaxation and improved sleep — in the absence of measurable pain score reduction, which may partly explain the dissociation between high overall satisfaction and variable self-reported pain relief.

## Discussion

The present prospective observational study found no evidence of clinically meaningful worsening of dystonia severity at the group level following conventional TENS use, as assessed by the TWSTRS total score against a predefined exploratory safety threshold. Although this study was not designed to formally demonstrate analgesic or motor efficacy, our findings indicate exploratory evidence suggesting pain improvement, particularly captured by the TWSTRS pain subscore, together with good tolerability and high patient acceptability.

### Safety of sensory electrical stimulation in CD

Demonstrating that TENS does not aggravate dystonia is a critical prerequisite before considering any neuromodulatory intervention in a disorder characterized by abnormal sensorimotor integration and maladaptive plasticity (4, 14–16). In our cohort, the mean change in the TWSTRS total score remained well above the exploratory −8-point safety threshold at the group level, although two individual patients fell below this threshold. These findings are particularly reassuring in the context of abnormal homeostatic plasticity described in dystonia (4, 14–16), suggesting that conventional sensory-level stimulation does not destabilize motor networks despite underlying inhibitory dysfunction.

Prior to the present study, evidence regarding the safety of cutaneous electrical stimulation in CD was limited. Early exploratory work suggested that selective sensory or electrical stimulation could transiently modulate dystonic activity without clinical worsening (17). More recently, high-frequency somatosensory stimulation revealed abnormal cortical plasticity in dystonia without inducing any clinical deterioration (18). Our results extend these observations by providing prospective safety data in a real-world clinical setting. Beyond its clinical implications, this study may be viewed as a proof-of-concept demonstration that sustained peripheral sensory stimulation can be applied safely in a disorder characterized by maladaptive sensorimotor plasticity.

### Analgesic effects: preliminary clinical signals

While no statistically significant reduction in pain intensity was observed using the numeric PNRS, a modest improvement was observed in the TWSTRS pain subscore. This discrepancy may simply reflect differences in the constructs measured by the two scales, as the TWSTRS pain subscore integrates current, best, and worst pain over the preceding week, potentially providing greater sensitivity to fluctuations in small samples (4).

Because TENS delivers a salient sensory stimulus and pain was assessed by patient self-report, a placebo contribution to the observed pain-related and Severity signals cannot be excluded in this open-label, non-sham-controlled design; these signals should not be interpreted as evidence of a specific analgesic effect of TENS.

These findings should be considered exploratory. The small sample size, heterogeneity of pain mechanisms, open-label design, and absence of a sham-control group substantially limit interpretation. Nevertheless, the observed trend toward improvement in TWSTRS pain scores, together with high patient satisfaction and continued daily use, suggests that some patients may perceive a clinical benefit. The wide standard deviation in self-reported pain relief (29.5 ± 20.6%) reflects marked inter-individual variability, suggesting that response to TENS may depend on pain subtype, underlying mechanism, or individual sensory processing.

### Pathophysiological considerations

Several mechanisms may account for the potential effects of TENS in painful CD. High-frequency TENS is thought to induce segmental inhibition of nociceptive transmission through activation of large-diameter afferent fibers, consistent with gate-control mechanisms, and to reduce central and peripheral sensitization (6, 7). At the cortical level, sensory electrical stimulation has been shown to modulate sensorimotor excitability. In CD, repetitive sensory stimulation can normalize somatosensory temporal discrimination and enhance inhibitory circuitry (19). Within this framework, TENS could act as a form of continuous sensory input analogous to a sustained sensory trick, with repeated stimulation potentially engaging similar circuits and contributing to both analgesia and subtle motor modulation (20–24).

### Interaction with BoNT therapy

Most patients included in this study were treated with BoNT, the first-line therapy for CD (15). The present findings suggest that TENS can be readily integrated into the management of patients with painful CD receiving routine care. However, interpretation of pain-related outcomes requires caution, as a potential confounding effect of BoNT cannot be completely excluded. Future randomized sham-controlled studies should investigate the specific contribution of TENS, its interaction with BoNT and physiotherapy, and the optimal timing of TENS use within the BoNT injection cycle.

A sensitivity analysis restricted to patients with an unchanged BoNT dose between T1 and T2 (plus the non-BoNT patients; *n* = 9) yielded results consistent with the full cohort, suggesting that dose adjustment alone does not explain the absence of a safety signal; however, this observational, non-factorial design cannot fully separate a TENS-specific effect from the fluctuating clinical effect of concurrent BoNT therapy. Given the small size (*n* = 9) and *post hoc* nature of this subgroup, it should be regarded as a sensitivity analysis pointing in a similar direction as the primary analysis, not as evidence that BoNT-related confounding has been excluded.

## Limitations

Several limitations must be acknowledged. First, the small sample size limits statistical power and generalizability. Second, the open-label design and absence of a sham group preclude firm conclusions regarding the specific efficacy of TENS beyond placebo effects. Although assessments were performed at comparable time points within the BoNT cycle, confounding cannot be fully excluded. Similarly, physiotherapy was used by a substantial proportion of participants. Finally, cervical pain in dystonia is frequently multifactorial, and coexisting musculoskeletal conditions may have reduced the ability to detect a specific TENS effect. Adherence was assessed through patient self-report, which may overestimate actual daily use.

Concomitant non-BoNT therapies (e.g., physiotherapy, medication) were not held constant between T1 and T2 by protocol and cannot be fully excluded as confounders of the exploratory pain-related findings, although BoNT dose and muscle selection were verified and a dose-stable sensitivity analysis yielded consistent results. Given the very small sample size (*n* = 15), all descriptive and nominally significant trends reported here—including the TWSTRS Severity change—should be regarded as preliminary and hypothesis-generating rather than confirmatory. The predefined −8-point exploratory safety threshold was extrapolated from published estimates of the minimal clinically important improvement because no validated threshold for clinically meaningful worsening exists for the TWSTRS. Although this represents a pragmatic and conservative approach, the assumption remains unvalidated and should be confirmed in future studies. A group-level mean and its confidence interval can conceal individual variability; for this reason, we also report the two individual patients whose T1–T2 change fell below this exploratory threshold.

## Conclusion

Overall, these findings support the feasibility of conventional TENS in painful cervical dystonia and provide reassurance regarding its short-term safety. Larger randomized sham-controlled studies are now warranted to confirm these preliminary observations, determine its analgesic efficacy, and further explore its potential role as a tool for investigating sensorimotor processing in cervical dystonia.

## Data Availability

The raw data supporting the conclusions of this article will be made available by the authors upon justified request.

## References

[1] Charles PD Adler CH Stacy M Comella C Jankovic J Manack Adams A . Cervical dystonia and pain: characteristics and treatment patterns from CD PROBE (cervical dystonia patient registry for observation of OnabotulinumtoxinA efficacy). J Neurol. (2014) 261:1309–19. doi: 10.1007/s00415-014-7343-6 PMC4098041 24752807

[2] Maione R. Formica C. Quartarone A. Lo Buono V. The impact of non-motor symptoms on quality of life in cervical dystonia. J Clin Med (2023);12:4663. doi: 10.3390/jcm12144663, 37510780 PMC10380526

[3] Charles PD Manack Adams A Davis T Bradley K Schwartz M Brin MF . Neck pain and cervical dystonia: treatment outcomes from CD PROBE (cervical dystonia patient registry for observation of OnabotulinumtoxinA efficacy). Pain Pract. (2016) 16:1073–82. doi: 10.1111/papr.12408, 26910788

[4] Brodoehl S. Wagner F. Prell T. Klingner C. Witte O. W. Günther A. Cause or effect: altered brain and network activity in cervical dystonia is partially normalized by botulinum toxin treatment. Neuroimage Clin. (2019);22:101792. doi: 10.1016/j.nicl.2019.101792 30928809 PMC6444302

[5] Viderman D. Nabidollayeva F. Aubakirova M. Sadir N. Tapinova K. Tankacheyev R. The impact of transcutaneous electrical nerve stimulation (TENS) on acute pain and other postoperative outcomes: a systematic review with meta-analysis. J Clin Med (2024);13:427. doi: 10.3390/jcm13020427 PMC10816585 38256561

[6] Johnson M. I. Paley C. A. Jones G. Mulvey M. R. Wittkopf P. G. Efficacy and safety of transcutaneous electrical nerve stimulation (TENS) for acute and chronic pain in adults: a systematic review and meta-analysis of 381 studies (the meta-TENS study). BMJ Open (2022);12:e051073. doi: 10.1136/bmjopen-2021-051073 PMC8845179 35144946

[7] DeJesus BM Rodrigues IKL Azevedo-Santos IF DeSantana JM. Effect of transcutaneous electrical nerve stimulation on pain-related quantitative sensory tests in chronic musculoskeletal pain and acute experimental pain: systematic review and meta-analysis. J Pain. (2023) 24:1337–82. doi: 10.1016/j.jpain.2023.03.014, 37030583

[8] Boyce MJ Canning CG Mahant N Morris J Latimer J Fung VSC. The Toronto Western spasmodic torticollis rating scale: reliability in neurologists and physiotherapists. Parkinsonism Relat Disord. (2012) 18:635–7. doi: 10.1016/j.parkreldis.2012.02.007, 22405838

[9] Cano SJ Hobart JC Fitzpatrick R Bhatia K Thompson AJ Warner TT. Patient-based outcomes of cervical dystonia: a review of rating scales. Mov Disord Off J Mov Disord Soc. (2004) 19:1054–9. doi: 10.1002/mds.20055, 15372595

[10] Kaji R Osawa M Yanagisawa N. Inter-rater reliability while using the Toronto Western spasmodic torticollis rating scale (TWSTRS) in patients with cervical dystonia. Brain Nerve. (2009) 61:65–71. 19177808

[11] Dworkin RH Turk DC Farrar JT Haythornthwaite JA Jensen MP Katz NP . Core outcome measures for chronic pain clinical trials: IMMPACT recommendations. Pain. (2005) 113:9–19. doi: 10.1016/j.pain.2004.09.012, 15621359

[12] Dashtipour K Mari Z Jankovic J Adler CH Schwartz M Brin MF. Minimal clinically important change in patients with cervical dystonia: results from the CD PROBE study. J Neurol Sci. (2019) 405:116413. doi: 10.1016/j.jns.2019.07.031, 31494402

[13] Espay AJ Trosch R Suarez G Johnson J Marchese D Comella C. Minimal clinically important change in the Toronto Western spasmodic torticollis rating scale. Parkinsonism Relat Disord. (2018) 52:94–7. doi: 10.1016/j.parkreldis.2018.03.002, 29530726

[14] Corp DT Joutsa J Darby RR Delnooz CCS van de Warrenburg BPC Cooke D Network localization of cervical dystonia based on causal brain lesions. Brain (2019);142:1660–1674. doi: 10.1093/brain/awz112 PMC6536848 31099831

[15] Albanese A Di Giovanni M Lalli S. Dystonia: diagnosis and management. Eur J Neurol. (2019) 26:5–17. doi: 10.1111/ene.13762, 30035844

[16] Albanese A Bhatia KP Fung VSC Hallett M Jankovic J Klein C Definition and classification of dystonia. Mov Disord (2025);40:1248–1259. doi: 10.1002/mds.30220 PMC12273609 40326714

[17] Leis AA Dimitrijevic MR Delapasse JS Sharkey PC. Modification of cervical dystonia by selective sensory stimulation. J Neurol Sci. (1992) 110:79–89. doi: 10.1016/0022-510x(92)90013-b, 1506873

[18] Erro R Rocchi L Antelmi E Liguori R Tinazzi M Berardelli A . High frequency somatosensory stimulation in dystonia: evidence fordefective inhibitory plasticity. Mov Disord. (2018) 33:1902–9. doi: 10.1002/mds.27470, 30376603

[19] Erro R Antelmi E Bhatia KP Latorre A Tinazzi M Berardelli A . Reversal of temporal discrimination in cervical dystonia after low-frequency sensory stimulation. Mov Disord. (2021) 36:761–6. doi: 10.1002/mds.28369, 33159823

[20] Rocchi L. Latorre A. Menozzi E. Rispoli V. Rothwell JC Berardelli A. Amelioration of focal hand dystonia via low-frequency repetitive somatosensory stimulation. Mov Disord (2024);39:2220–2229. doi: 10.1002/mds.30011 PMC11657077 39254362

[21] Naumann M Magyar-Lehmann S Reiners K Erbguth F Leenders KL. Sensory tricks in cervical dystonia: perceptual dysbalance of parietal cortex modulates frontal motor programming. Ann Neurol. (2000) 47:322–8. 10716251

[22] Cai N. Q. Shi W. X. Chen R. K. Chen B. L. Li Y. R. Wang N. Cerebral-cerebellar cortical activity and connectivity underlying sensory trick in cervical dystonia. Ann Clin Transl Neurol (2024);11:2633–2644. doi: 10.1002/acn3.52177 PMC11514925 39152615

[23] Sarasso E Agosta F Piramide N Bianchi F Butera C Gatti R . Sensory trick phenomenon in cervical dystonia: a functional MRI study. J Neurol. (2020) 267:1103–15. doi: 10.1007/s00415-019-09683-5, 31897600

[24] Manzo N. Leodori G. Ruocco G. Belvisi D. Merchant SHI Fabbrini G. Cortical mechanisms of sensory trick in cervical dystonia. Neuroimage Clin (2023);37:103348. doi: 10.1016/j.nicl.2023.103348 PMC9950946 36791488

