# Supplementary material for: Conventional transcutaneous electrical nerve stimulation shows no short-term safety signal in painful cervical dystonia: a pilot prospective study
Source: Front Neurol. 2026 Oct 7;17:1909821. doi: 10.3389/fneur.2026.1909821 (PMC13683213; doi:10.3389/fneur.2026.1909821)
Supplement: Supplementary file 1 [file Table_1.DOCX]

**Supplementary Material: Tables**

**Supplementary Table 1.** TENS use pattern, tolerability, and outcomes in patients who completed the study (n = 15).

| **Variable** | **n = 15** |
| --- | --- |
| **TENS usage** | |
| Daily TENS use, min/day — Mean ± SD [range] | 72.3 ± 42.3 [30–150] |
| Onset of pain relief, min — Mean ± SD [range] | 5.3 ± 9.1 [0–30] |
| Residual analgesic effect after removal, n (%) | 12 (80) |
| Self-reported pain relief, % — Mean ± SD [range] | 29.5 ± 20.6 [0–60] |
| **Outcomes** | |
| Overall satisfaction and willingness to continue, n (%) | 12 (80) |
| TENS continuation at end of follow-up, n (%) | 12 (80) |
| TENS discontinuation, n (%) | 3 (20) |
| Reason: insufficient pain relief, n (%) | 2 (13.3) |
| Reason: cervical pain predominantly arthritic, n (%) | 1 (6.7) |
| **Safety** | |
| Any adverse event, n (%) | 6 (40) |
| Electrode detachment with local discomfort | 3 (20) |
| Occasional electrical shocks at electrode detachment | 2 (13.3) |
| Shoulder/arm fatigue → discontinuation | 1 (6.7) |
| Allergic reaction | 0 |

*TENS, transcutaneous electrical nerve stimulation. All adverse events were mild and transient. Percentages for discontinuation reasons are calculated among the 3 patients who discontinued TENS.*

**Supplementary Table 2.** Individual patient TENS data (n = 15).

| **Patient** | **Daily use** | **Onset (min)** | **Residual effect** | **Relief (%)** | **Continuation / reason for stop** | **Adverse events** |
| --- | --- | --- | --- | --- | --- | --- |
| 1 | 60 min/d | 0 | No | 0 | No — pain control with physiotherapy alone | None |
| 2 | 120 min/d | 30 | Yes (1 h) | 50 | Yes | None |
| 3 | 40 min/d | 0 | Yes (1 h) | 20 | Yes | None |
| 4 | 30 min/d | 0 | Yes (30 min) | 60 | Yes | None |
| 5 | 120 min/d | 0 | Yes (1 h) | 20 | Yes | None |
| 6 | 30 min/d | 0 | Yes (few h) | 60 | Yes | None |
| 7 | 30 min/d | 10 | Yes (few h) | 2 | No — shoulder/arm fatigue → discontinuation | Shoulder/arm fatigue 2–3 days post-session |
| 8 | 30 min/d | 10 | Yes (few h) | 30 | Yes | Electrode detachment + local pain → resolved with gel |
| 9 | 60 min/d | 10 | Yes (few h) | 20 | Yes | None |
| 10 | 2 h/3 days | 0 | No | 0 | No — insufficient pain relief | Arm pain (electrode misplacement) → resolved after repositioning |
| 11 | 30 min/d | 0 | Yes (1 h) | 40 | Yes | Electrode detachment + local pain → resolved with gel |
| 12 | 90 min/d | 0 | Yes (few h) | 25 | Yes | None |
| 13 | 45 min/d | 0 | Yes (few h) | 50 | Yes | None |
| 14 | 120 min/d | 0 | Yes (few h) | 20 | Yes | Occasional electrical shocks at electrode detachment |
| 15 | 120 min/d | 20 | Yes (few h) | 45 | Yes | Occasional electrical shocks at electrode detachment |

*h, hours; min, minutes. 'Residual effect' refers to analgesic benefit persisting after TENS removal. Pain relief (%) is self-reported by the patient at follow-up (T2). Continuation refers to willingness to continue TENS use at end of follow-up.*
